# The socialization of modality capital in sign language ecologies: A classroom example

**DOI:** 10.3389/fpsyg.2022.934649

**Published:** 2022-10-28

**Authors:** Jenny L. Singleton, Peter K. Crume

**Affiliations:** ^1^Department of Linguistics, Stony Brook University, Stony Brook, NY, United States; ^2^Department of Learning Sciences, Georgia State University, Atlanta, GA, United States

**Keywords:** deaf, gaze behavior, teacher-student interaction, attention, socialization, modality capital

## Abstract

Gaze behavior is an important component of children’s language, cognitive, and sociocultural development. This is especially true for young deaf children acquiring a signed language—if they are not looking at the language model, they are not getting linguistic input. Deaf caregivers engage their deaf infants and toddlers using visual and tactile strategies to draw in, support, and promote their child’s visual attention; we argue that these caregiver actions create a developmental niche that establishes the *visual modality capital* their child needs for successful sign language learning. But most deaf children do not have deaf signing parents (reportedly over 90%) and they will need to rely on adult signing teachers if they are to acquire a signed language at an early age. This study examines classroom interactions between a Deaf teacher, her teacher’s aide, and six deaf preschoolers to document the teachers’ “everyday practices” as they socialize the gaze behavior of these children. Utilizing a detailed behavioral and linguistic analysis of two video-recorded book-sharing contexts, we present data summarizing the teacher’s attention-getting actions directed toward the children and the discourse-embedded cues that signal the teacher’s expectations for student participation in the signed conversation. We observed that the teacher’s behaviors differed according to the parent status of the deaf preschooler (Deaf parents vs. hearing parents) suggesting that Deaf children of Deaf parents arrive to the preschool classroom with well-developed self-regulation of their attention or gaze. The teachers also used more physical and explicit cueing with the deaf children of hearing parents—possibly to promote their ability to leverage the visual modality for sign language acquisition. We situate these socialization patterns within a framework that integrates notions of intuitive or indigenous practices, developmental niche, and modality capital. Implications for early childhood deaf education are also discussed.

## Introduction

Deaf caregivers who sign fluently actively use visual and tactile strategies to draw in, support, and promote their deaf infant’s visual attention skills (e.g., Spencer et al., 1992; Swisher, 1992; Waxman and Spencer, 1997; Spencer and Harris, 2006; Pizer et al., 2011). These behaviors include physical contact and waving, adjusting interlocutor proxemics to ensure gaze connection, signing bigger and with repetition, and proto-turntaking actions. Taken together, these caregiver behaviors appear to socialize *visual modality capital*; that is, ensuring that the visual modality can be leveraged for language input. Adults who engage in these behaviors enable a deaf child to learn where to look for, and even anticipate, the source of signed linguistic information. More specifically, the child learns that (a) following their caregiver’s gaze will lead to meaningful information, (b) shifting their gaze back to the caregiver after object exploration will provide linguistic information, (c) gaze shifting will enable them to follow multi-party signed conversation, (d) effective visual access means no visual obstruction should be blocking the source of linguistic information, (e) movement and visual cues (perhaps later shifting into more linguistic cues) can serve as turn taking regulators [see Horton and Singleton (2022), in this volume]. With this modality capital leveraged, a young deaf child is thus primed to acquire a language that is organized in the visual modality.

It is important to point out that modality capital can be socialized in either the auditory or visual modality. Much of the work on speech prosody, infant-directed speech patterns, speech-specific sensitivities, may be evidence for auditory modality capital in that caregiver practices can be leveraged to support spoken language acquisition (Newport et al., 1977; Grieser and Kuhl, 1988; Fernald et al., 1989). We do not assume that modality capital is acquired similarly in the auditory and visual modalities. For hearing-seeing children, the simultaneously experienced auditory (speech) and visual information presumably must be integrated to support spoken language acquisition. Auditory modality capital may be considered universal for hearing infants, even though natural variation in caregiver speech patterns occurs [see Ochs and Schieffelin (1984), Bakeman et al. (1990), Rogoff et al. (1993), and Chavajay and Rogoff (1999), for discussions of variation in caregiver speech patterns and ideologies about children and their language learning].

By contrast, deaf children are likely unable to leverage auditory modality capital for language acquisition. They will need to rely on visual information coming from multiple sources to build visual modality capital; this means they will need to learn where to look and how to integrate linguistic information from signing caregivers with objects that are visually present in the world. The extent to which deaf caregivers explicitly socialize deaf infants, creating a developmental niche (Super and Harkness, 1986, 2002) that helps them leverage visual modality capital for sign language acquisition, will be discussed in greater detail below. In her comprehensive review of the ways that social contexts support and shape language development, Hoff (2006) does not consider how language modality factors into the ecology where language acquisition takes place. We maintain that the human solution of leveraging visual modality capital for sign language acquisition may also be a “reliable result of the mental processes set in motion when the child meets the social and linguistic world” (Hoff, 2006, p. 78).

### Importance of attention and gaze

Joint attention, where an adult and infant jointly attend to the same object, is viewed by many developmental researchers to be a key psychological process and is argued to be critical for developing basic socio-cognitive understanding and language in the auditory modality (Tomasello and Farrar, 1986; Bornstein, 1990; Adamson and Bakeman, 1991; Baldwin, 1995; Tomasello, 1995, 1999; Carpenter et al., 1998; Brooks and Meltzoff, 2002, 2005, 2008; Mundy, 2003). An infant first “learns to” gaze-follow and then “learns from” gaze-following as the social-cognitive component becomes better established (Vaughan van Hecke and Mundy, 2007, p. 40). The capacity to self-regulate one’s own visual attention also serves as one of the earliest components of the Executive Functions to come “online” (Anderson, 2002). According to Posner and Rothbart (2000, 2007), there are three stages of orienting attention. First, an individual must disengage from what they are presently looking at, then they must shift their attention to the new location, and finally they engage their attention to the new target. While there are early developing capacities in self-regulation of attention around ages 9–12 months (Ruff and Rothbart, 1996), the period between 12 and 36 months marks a significant advance in the child’s self-regulatory abilities (Bronson, 2000). Researchers have described both exogenous (e.g., a caregiver’s voice, a loud noise, or a flashing light) and endogenous factors (e.g., self-interest in a toy) that contribute to the process of orienting our attention. It is important to recognize that a child’s developing capacity to engage in mutual and joint attention, insofar as they integrate what they see and what they hear, is shaped by both maturation and environmental/interactional processes (Rothbart et al., 1990; Ruff and Rothbart, 1996; Mundy and Sheinkopf, 1998; Posner and Rothbart, 2000).

Studies involving hearing children have shown that eye gaze serves as an important window into cognitive functioning. For example, children who are later diagnosed with autism spectrum disorder are found as young children to exhibit gaze behaviors that differ from neurotypical children (Dawson et al., 1998; Baron-Cohen, 2000; Mundy et al., 2000; Adamson et al., 2009; Klin et al., 2009). Children with Down Syndrome are slower to hit developmental milestones in gaze following (Adamson et al., 2009). Children with Attention Deficit Disorder also show atypical patterns of development of attention/gaze [see Ruff and Rothbart (1996), for review]. In typically developing children under the age of one, the capacity to follow an adult’s shift in gaze appears to be a significant correlate of early spoken language vocabulary acquisition (Mundy et al., 1995; Carpenter et al., 1998; Morales et al., 2000; Meltzoff and Brooks, 2008). Lastly, gaze behavior and proxemics can also vary or be influenced by local gaze norms used by hearing individuals within different cultural communities (Chavajay and Rogoff, 1999; Gaskins and Paradise, 2010; de León, 2011; Haviland, 2020; Horton et al., under review^1^).

In this study, we examine the *socialization of gaze behavior* among deaf children. We first explore how deaf caregivers establish linguistic and modality-based practices to promote their children’s development of attention and successful visual language acquisition. We then investigate how deaf teachers take on this same task within the context of their preschool classroom.

### Theoretical framework for socializing gaze and attention

As we explore further how adults socialize deaf children’s attention and gaze behavior, we shall first outline some theoretical orientations that frame our interpretation of this developmental process. First, we look at the social engagement behaviors initiated by adults and directed toward children as part of a larger system of parenting beliefs and practices, communication, and socio-cultural interaction patterns within a community. Caregivers possess certain indigenous knowledge systems or intuitive parenting practices (Papoušek and Papoušek, 1987), use culturally relevant artifacts, and hold certain beliefs about children’s capacities, all of which form what Super and Harkness (1986, 2002) call a “developmental niche.” Within this niche, caregivers guide their children, scaffolding their behaviors, and support their development as full participants in their family and community (Rogoff, 1990, 2003; Rogoff et al., 1993; Chavajay and Rogoff, 1999).

Beyond the social interaction perspective, we also situate gaze behavior within a developmental and dynamic cognitive system (Corina and Singleton, 2009). Control of one’s attention allocation is part of a larger cognitive system regulated by the executive functions of the brain. Self-regulation requires both active attending as well as inhibition (i.e., suppressing one’s interest in an attractive object in response to a caregiver’s bid for attention). As a child builds capacities in basic attention regulation, one sees growth in more “higher order” cognitive processes such as working memory, planning, and cognitive flexibility (Ruff and Rothbart, 1996). It is important to note that all children, hearing or deaf, are visually oriented and develop gaze-following behavior that is eventually self-regulated. What is unique about being raised in deaf, sign language-using families, is that attracting, maintaining, and directing an infant’s visual attention is essential for visual language communication to take place. The literature on deaf caregivers’ visual engagement patterns suggests strongly that their young children are being socialized to attend in unique ways. Deaf caregivers often create a developmental niche that appears to capitalize upon the visual modality and results in the shaping of an infant’s attentional capacities. We argue that they are intuitively building “modality capital,” through which caregiver-child interactions—replete with attention-shifting and linguistic demands—become a synchronous and natural experience.

### Socialization of deaf children’s visual modality capital

The social and communicative interactions between Deaf caregivers and their deaf children have been studied across many cultural contexts [see Spencer and Harris (2006), for a review] including the United States (Erting et al., 1990/1994; Spencer et al., 1992; Swisher, 1992; Waxman and Spencer, 1997; Koester et al., 1998; Lieberman et al., 2011, 2014; Pizer et al., 2011), Canada (Jamieson, 1994), the United Kingdom (Harris et al., 1989; Ackerman et al., 1990; Smith and Sutton-Spence, 2005; Guarinello et al., 2006), Australia (Mohay et al., 1998), Belgium (Loots and Devise, 2003; Loots et al., 2005), and Japan (Masataka, 1992). Many deaf caregivers scaffold visual modality capital by engaging their young children in particular ways that attract and maintain their visual attention ensuring that the child is able to see the signed language input the caregiver provides. Some examples of this visual “attunement” include producing signs within child’s visual field, pausing their signing until the infant is looking, moving objects closer to the caregiver’s face, using more exaggerated facial expressions, imparting rhythmicity in a sign’s movement, and use of visual attention-getting behaviors like waving at or tapping the child (Pizer et al., 2011). Some caregivers also use tactile, vocal, and kinesthetic stimulation (Harris et al., 1989; Koester et al., 1998, 2004). Deaf caregivers also appear to use shorter phrases and repetition in their signing (Spencer and Harris, 2006). This strategy enables them to capitalize on the potentially brief window of opportunity of mutual connected eye gaze and provides multiple opportunities for the child to make associations between the visual referent and the signed form. Many of these caregiver behaviors decrease over time as the infant increases their self-regulation of attention (vis-à-vis accrued modality capital) as well as understands that the tapping or waving signal means “look to the caregiver” for language. Eventually, the child will anticipate the appropriate time to look-to-caregiver, relying upon linguistic devices and turn-taking cues present in the discourse, rather than being physically tapped by the caregiver.

From the perspective of the child, we know that deaf infants born to deaf (DoD) families show early control over gaze following and gaze-shifting compared to non-signing hearing infants (Brooks et al., 2019). Lieberman et al. (2014) investigated Deaf mothers and their children engaging in book-sharing activities. They observed that even by the age of 2, the DoD toddlers more frequently shifted their eye gaze back and forth between the caregiver and the book as compared to deaf children of hearing parents. We also know that compared to deaf children of hearing parents (DoH), DoD engage in more spontaneous looking to their caregiver (which requires inhibiting one’s attention from an interesting object and shifting one’s gaze to the caregiver) (Harris and Mohay, 1997). This is not to say that hearing caregivers do not engage in modality capital promoting behaviors with their deaf child, but the primary finding from accumulated observational research is that there is more variability in hearing parents’ attention-getting strategy use, greater asynchrony in their timing of sign production, and their “bouts” of joint attention with their deaf child are shorter, thereby leaving a narrower window of language learning opportunity (Spencer and Harris, 2006). Furthermore, Prezbindowski et al. (1998) contend that deaf children of hearing caregivers exhibit atypicality in their regulation of attention “…long before they exhibit noticeable language delays” (386).

To summarize, research on caregiver–child interaction in infancy and toddlerhood suggests that deaf children born to deaf families are being socialized into a visual language community through a set of everyday caregiver behaviors that ensure the child will develop visual modality capital. For deaf children born to hearing parents (reportedly over 90% of the deaf population, Holcomb, 2013), however, the early childhood education classroom, possibly with a deaf signing teacher, may be the first “caregiver-like” context in which they are exposed to the kinds of systematic socialization of visual modality capital that has been so well-documented in deaf–deaf family dyads (Singleton and Morgan, 2006; Singleton and Meier, 2021). Moreover, expectations for the child’s classroom behavior (e.g., sitting still in a preschool class) will also require the child to increase in inhibitory control, sustained attention, and shifts in attention (Ruff and Rothbart, 1996).

### Teachers as socializing agents of visual modality capital

There are a few classroom studies where teachers’ use of visually based socialization practices with deaf students is documented. For example, Mather (1987) found that in teacher-led group interaction, signing teachers use three types of eye gaze signals to convey information about their intended addressee. *Group-indicating* gaze employs a “smooth arc-like” glance toward the group and indicates that the teacher’s question or comment is intended for all group members. Similarly, *audience gaze* conveys that the entire group is the intended addressee, but in this case, a teacher selects a midpoint of the group to affix her gaze, rather than the swoop of the group-indicating gaze. *Individual gaze* is directed at one child and conveys to other members of the group that it is not their turn; instead, that the floor is to be held by the specific addressee. In sum, students in signing classrooms may use the teacher’s eye gaze cues to learn when they are being addressed and whether it is appropriate to make a bid for the teacher’s attention.

Eye gaze signals convey important discourse cues to the conversational partner. Mather and Thibeault (2000) explain that signers use gaze, along with the creation of a “surrogate” signing space and head/shoulder tilts, to convey “constructed dialogue.” Such embodied “role shifts” tell the other signer that you are not speaking directly to them, but rather you are becoming another character, similar to “reporting speech.” This way the addressee understands that the storyteller is no longer in narrator mode but is constructing the dialogue in the story. Hearing children can rely upon auditory cues such as changes in voice quality and other paralinguistic features to identify which character the narrator has become. In contrast, deaf children rely upon the eye gaze behavior and body posture of the storyteller to follow the discourse shifts (Mather and Thibeault, 2000).

To investigate the visual engagement patterns of a deaf teacher interacting with deaf (*n* = 2) and hearing (*n* = 2) preschoolers as they engaged in different play contexts (play dough and dramatic play), DeLuzio and Girolametto (2006) adapted Koester et al.’s (1998) coding system for documenting caregiver’s attention-eliciting behaviors. While no differences across play contexts were found, the deaf educator relied heavily upon tactile and visual attention-getting strategies with her 3- and 4-year-old students. The authors suggest that the educator may also have responded differently to the hearing status of the child, but they did not provide corresponding data broken down with respect to this issue.

Smith and Ramsey (2004) looked at older deaf students in fifth grade and analyzed their classroom interactions with a deaf teacher. While the focus of this study was more on instructional conversation discourse features, there were some documented patterns of gaze, non-manual markers, and discourse-embedded cues that were deployed by the teacher to control conversation flow. The teacher was also persistent in her attempts to get deaf students engaged and frequently checked their comprehension (often through a subtle non-manual marker). Smith and Ramsey also noted that the students in the class used hand-raising and hand-waving to gain the teacher’s attention (54).

Departing from a focus on the actions of deaf teachers, Lieberman (2015) documented the attention-getting actions produced by seven deaf native ASL signing toddlers during their classroom interactions with deaf peers and their teachers. Briefly, Lieberman shows compelling evidence that young deaf toddlers are already capable of using attention-getting strategies in their signed interactions with their classmates. We will pick up again on Lieberman’s analysis in the Section “Discussion.”

In sum, a young deaf child immersed in a visual language ecology (i.e., a developmental niche) learns to rely on sophisticated and complex eye gaze signals in order to leverage the visual modality and gain access to linguistic input (signed language) and acquire the social interaction norms for visual language exchanges. Apart from the aforementioned studies, the research literature has not documented classroom interaction processes to a level that will help us understand better “what works” in deaf educational settings and how particular instructional strategies may be more effective than others in building deaf children’s visual modality capital.

## Materials and methods

For this study, we conducted a detailed naturalistic observation of deaf teachers in early childhood deaf education classrooms, across two different interaction contexts, to document the kinds of teacher practices that were used to gain and direct deaf preschooler’s visual attention. By examining the type of teacher strategy, as well as to whom (Deaf child of Deaf parents, or deaf child of hearing parents) a particular strategy was directed, we could document teacher’s behaviors that appear to socialize a deaf child’s visual modality capital.

### Participants

The study includes one teacher and one teacher’s aide, both deaf and highly fluent in American Sign Language (ASL). The teacher was nominated for the study by the principal of the residential school for the deaf for being an outstanding ASL language model at the preschool level. All six children in this selected preschool classroom have profound or severe-to-profound hearing loss (see Table 1). Child 1 (male, age 4;8), Child 4 (male, age 4;6), and Child 6 (male, age 5;0) had Deaf ASL-using parents. The teacher reported to us that all of these Deaf parents are fluent in ASL based on her firsthand experience interacting with them. Child 2 (female, age 5;5), Child 3 (male, age 5;8), and Child 5 (female, age 5;10) had hearing parents or guardians, whom the teacher reported has minimal ASL signing skills. While we recognize that parent hearing status is not always a proxy for ASL fluency, in the specific case of this study, we felt comfortable using the DoD and DoH terminology to reflect these children’s early signing experience and their potential level of ASL fluency. We note that the three DoD were on average, younger than the three DoH children in this Pre-K classroom. Child 3 (DoH) has a cochlear implant on the right side that was not in use at the time of the study. Child 5 (DoH) had only been in the classroom for a few weeks, while the other students had been enrolled at least since the start of the school year in this program, which was a few months before our observation.

**TABLE 1:** Child characteristics.

Child	Gender	Age	Hearing status		Ethnicity
			Child’s	Parents’	
1	Male	4;8	Profound	Deaf	White
2	Female	5;5	Severe to profound	Hearing	White
3	Male	5;8	Profound	Hearing	White
4	Male	4;6	Severe to profound	Deaf	White
5	Female	5;10	Profound	Hearing	Black
6	Male	5;0	Profound	Deaf	White

### Context: Bilingual American Sign Language/English preschool for deaf children

To examine these socializing practices, we analyzed video-recorded data that captured natural interactions in preschool classrooms between deaf teachers and deaf children who are 4-to-5 years old. The selected preschool is part of a residential school for deaf children adopting a bilingual, bicultural approach to communication. The school uses two languages for communication: ASL and English (primarily through the written form, although some students also receive some spoken English instruction during the day). The data for this study are drawn from a larger collection (18 h) of video-recordings from multiple preschool and nursery school classrooms with deaf teachers at this site. The classroom interactions were recorded using a single video camera on a portable tripod during six visits over one semester. Different activities were recorded including group-based and individual activities involving several Deaf teachers. The video-recordings were collected by one co-author (Singleton, a hearing native ASL signer) and another investigator (a hearing, second language learner of ASL with very high fluency) after several observational visits without a camera so that the children would get used to their presence as observers. The second co-author (Crume, a hearing native ASL signer) joined the project at the coding and data analysis stage. The teachers were told that we were generally interested in classrooms where ASL is the language of instruction and that they should go about their normal routines. From the video-recordings, it is evident that the teachers and children went about their everyday classroom business; in the case of the selected episodes, the children and teachers were clearly used to the camera and researchers’ presence and did not look at the researchers during the episode.

### Episode selection

For the purpose of this study, we wanted to use two episodes of teacher-led group book-sharing sessions, from the same teacher. From the larger archive, we identified two episodes that met the following criteria: similar in length, had the same six students in attendance, and used the same book during the sharing activity. We targeted teacher-centered book-sharing sessions because these contexts require a high level of visual engagement and attention management (both teacher-directing and student self-regulating). In these episodes, the teacher is typically seated on the floor with the six children seated facing her in a semi-circle. The teacher must gain and maintain the children’s attention and the children must rapidly shift their gaze to other children as children take turns “holding the floor.” Additionally, the teacher directs children’s attention to a particular child, a book, or other visual media (such as a calendar). The children also vie for the teacher’s attention when attempting to bid for the floor.

The first selected episode is just over 20 min in length, and the second is closer to 16 min. In the first episode, the teacher introduces students to a particular storybook for the first time. In this activity, the teacher did not read the book verbatim, but instead lets students examine each page and offer their own comments about the story (“a picture walk”). There is minimal structure in this activity and students were free to respond when they had ideas to share. In the second episode, video-recorded 2 weeks later, the same teacher engages the same six children in a dramatic “roleplay” retelling of the same storybook used in the earlier picture walk episode. In this activity, the teacher assumes the role of the main character in the book and each student plays a specific animal character found in the story. The students appear familiar with the story because of prior teacher readings before this point in the data collection; they know the content of the book and their responses follow the actions their animal characters experienced in the story. This second episode also includes a deaf teacher’s aide seated on the floor just behind the semi-circle of students. We did not obtain information from the principal about the signing skills of the deaf Teacher’s Aide. Our informal impression based on reviewing video-recordings in the full archive is that she is a fluent signer of ASL.

The storybook, *Ask Mr. Bear* (Flack, 1932), was used in both video-recorded book-sharing activities. The book is about a boy who goes out looking for a birthday present for his mother. In his search, the boy meets different animals and asks them if they have anything to offer as a potential present (e.g., feathers, wool, milk, cream). As he meets each animal, the boy finds that he already possesses what each animal has to offer until he meets Mr. Bear who suggests that he give his mother a bear hug.

#### Context for episode 1 (picture walk)

In the first group activity, the picture walk, the teacher tries to connect the animal characters in the book with the students’ own experiences with animals. She opens the activity by discussing what students saw at a previous class field trip to the zoo. The teacher asks each student to recount his or her experience on the zoo trip, rapidly moving from one student to the next. In the middle of this sequence, the teacher stops at one student because she remembered that he did not go on the zoo field trip because he had his tonsils removed. The teacher uses this opportunity to discuss further the student’s experience being hospitalized, while encouraging the rest of the students to watch the conversation. After this sidebar with the zoo-absent student, the teacher resumes asking the other students about zoo animals. She subsequently asks the children to predict what animals they might see at an upcoming field trip to a farm. After the question and response activity about the farm animals, the teacher introduces the *Ask Mr. Bear* book to the students and explains that she wants their input about the story. However, the students are quite distracted, and it takes her a considerable amount of time to settle them down and focus on the main part of the book-sharing activity. After the teacher gains the students’ attention, she begins the picture walk activity. She subsequently shows the students each page, pointing to specific features in the illustrations, and asks students to share their thoughts. In the middle of the activity, a few students lose focus and begin to play and disregard the book-sharing activity. The teacher regains the attention of these students and encourages them to focus again on the picture walk activity. Once the students are resettled, she continues the picture walk until its completion. Table 2 provides an event breakdown and description of the picture walk book-sharing activity.

**TABLE 2:** Periods within episode 1–picture walk (total time: 21:33).

Period	Minutes	Description
Introduction	3:55	The teacher connects a previous zoo fieldtrip and an upcoming trip to a farm to prepare them for the Ask Mr. Bear book, which features several animals
Sidebar	2:01	The teacher interrupts her introduction to engage in a sidebar conversation with a student about his experience getting a tonsillectomy and uses this as a teachable moment for the class
Transition	1:43	The teacher prepares the students for the book sharing activity by providing instructions of the “Picture Walk” activity
Main activity (picture walk)	13:01	Students describe what they think is happening on each page of the book. The teacher scaffolds students’ learning by elaborating upon their responses
Refocus	0:53	During the middle of Picture Walk, the students become somewhat disengaged and the teacher redirects their attention back to the book

#### Context for episode 2 (role play)

The second group activity, the role play, occurred 2 weeks later. In the role play, the teacher displays a tray of props that includes a paper cut-out picture of each animal that appears in the book, an index card with character’s name, and a specific item relevant to each animal (e.g., wool for the sheep, feathers for the duck). The role play activity is obviously familiar to the students. The teacher begins the activity by stating it [the story] was the same as the other day. Immediately, several students get up from their sitting position in the semi-circle and crawl over to the prop tray and begin to grab props for a character they want. The teacher and aide have to get the attention of several students, encourage them to sit down, and assure them that they will each have their opportunity to select a character. Once the students are settled, the teacher asks each student which character they prefer and distributes the corresponding prop from the tray to each student. She then initiates the dramatic role play story retelling of *Ask Mr. Bear.* In the role play, the teacher assumes the main character role of the boy in the story and then engages each student as his/her specific character in the order they appear in the book. An event breakdown of the role play activity is detailed in Table 3.

**TABLE 3:** Periods within episode 2 (role play) (total time: 15:40).

Period	Minutes	Description
Introduction	1:55	The teacher and aide work to get the students settled and explain the upcoming activity
Distribution	3:40	The teacher distributes the props that the students will use during the storybook activity
Transition	0:45	The teacher and aide work to settle the children and begin the storybook activity
Main activity (roleplay)	9:20	The teacher tells the story by taking on the role of the main character while the students respond according to their assigned character in the book. The book text is not “signed aloud” word by word

### Coding procedure

Our coding procedure is an integration and modification of coding systems used by three different research groups in their analysis of classroom interactions involving deaf students (Mather, 1987, 1989; Mather and Thibeault, 2000; Smith and Ramsey, 2004; DeLuzio and Girolametto, 2006). Mather and colleague (Mather, 1987, 1989; Mather and Thibeault, 2000) analyzed preschool classroom interactions with deaf students and teachers and classified whether the teachers’ gaze was directed toward the entire group or toward an individual student. DeLuzio and Girolametto (2006) analyzed how teachers used visual and tactile strategies to gain or regain students’ attention in structured and unstructured educational contexts. Finally, Smith and Ramsey (2004) investigated classroom discourse practices, for example speaker roles and devices that maintain discourse coherence. Our adaptation combines and extends these authors’ research by including a range of attention-getting and attention-directing behaviors produced by the teachers, as well as how they manage the participation roles of the children in both structured and unstructured discourse settings. These specific actions are further detailed in the next section.

The coding of teacher’s language and actions was completed by both co-authors, who are both hearing, native ASL signers. As one independent measure of the co-authors’ ASL skills, both have earned national certification as sign language interpreters and possess many years of experience engaged in sign language-related research. Each co-author independently coded 25% of the other co-author’s coding to ensure coding accuracy. The very few coding discrepancies that occurred were resolved through discussion and resolution.

To be clear, the students’ attention actions (e.g., direction of eye gaze) are not directly analyzed in this coding scheme because, with the limitations of a single camera view that was trained primarily on the teacher (with the semi-circle of students in view), we could not reliably record the student’s gaze behaviors.

#### Attention actions and participation cues of the teacher

The teacher’s production of *Attention Actions* and *Participation Cues* emphasize different aspects of the socialization of children’s visual engagement (i.e., visual modality capital). Attention actions represent behaviors used by the adult to direct the eye gaze of the students, either toward the adult requesting the attention (i.e., toward self) or to another target, such as another adult, classmate, or resource. Participation cues represent the kind of scaffolding an adult produces within discourse that serves to maintain discourse cohesion (e.g., NOW, OK) but also implicitly conveys that “you should be looking at me”; also, these cues inform or shape the child’s behaviors with respect to appropriate participation in a visual language-using group interaction (e.g., WAIT, HOLD, YOUR-TURN). For example, in our observations, adults use participation cues to support students’ development of appropriate timing for turn-taking and cues about positioning themselves for successful visual engagement (e.g., sitting upright and ensuring no obstacles or people are obstructing the child’s view of the signer).

Attention actions produced by the teacher and aide are further divided into two types: *Attention-Gaining* actions and *Attention-Directing* actions. Attention-Gaining (AG) actions serve to attract the gaze of students toward the teacher. Attention-Directing (AD) actions attempt to re-direct the attention of a student to another adult, peer, or target object. Both types of attention actions include the same three categories of prompts used by the adult: linguistic, physical, and non-manual. Linguistic prompts are single signs or short phrases such as HEY! (hand-wave), PAY-ATTENTION, LOOK-AT-ME, LOOK-AT-HIM. These signs are produced within the visual field of the student(s). Physical prompts are light touches or taps on the child’s body (e.g., shoulder, arm, leg) or physical actions on an object (e.g., shaking an object to attract the child’s attention). Non-manual prompts are actions that include only the use of facial expressions or head/body behavior (e.g., tilting head) to draw the child’s attention toward the adult or another person (there is no co-occurring sign with the non-manual prompt). While it is not discussed specifically here, many of the linguistic prompts did co-occur with animated facial expression—this would be expected in the child-directed register that is being used by the teacher. Each AG and AD action is counted; for repeated signs (e.g., LOOK, LOOK, LOOK) each token is counted individually. A list of examples is provided in Table 4.

**TABLE 4:** Coding category descriptions: Teacher’s visual engagement actions.

Type	Category	Description	Examples of signs or behaviors
Attention-gaining	Linguistic prompt	Single signs or short phrases used within the visual field of student(s)	PAY-ATTENTION; LOOK-AT-ME; HEY! (Hand-wave); Calling child’s name (with namesign or fingerspelling)
	Physical prompt	Light tap or touch on the child when he/she is not attending to the teacher	Tapping, Nudging, Holding different part of body
	Non-manual prompt	Use of non-manual markers (without accompanying sign) in the visual field of the child	Facial expressions (e.g., raised eyebrows for “Well?” or pursed lips for “I’m waiting”); shoulder shrugs
Attention-directing	Linguistic prompt	Signs or short phrases used within the visual field of the student(s) in order to direct attention toward a person or object.	LOOK-AT-THIS (teacher-student-object); LOOK-AT-HIM/HER; re-directing point
	Physical prompt	Enhancing visual interest of the object to direct child’s attention toward it	Shaking an object (e.g., raising a book up and down) to attract the child’s attention to it.
	Non-manual prompt	Use of non-manual markers (without accompanying sign) in the visual field of the child	Head tilt and eye glances to direct child’s attention toward another person or object
Participation cues	Invite	Action or a statement that signals to student(s) that they should be attending, and may be encouraged to make a statement or ask a question	READY?; NEXT; a point to the person (finger or arm point), a head nod
	Directive	Authoritative comment with the intention of monitoring or altering the child’s undesirable (e.g., visually obstructed) position, behavior, or action	MOVE-FORWARD, MOVE-BACK, SIT-UP, SEE-CLEAR?
	Delay	Comment intended to get students to wait or postpone a specific request or comment	WAIT, WILL++, HOLD
	Refusal	Action produced in response to a child who is inappropriately bidding for the teacher’s attention. The teacher does not yield her attention to this interruption.	Not giving eye contact to a student who is bidding for attention; pushing down or holding the child’s hand

Participation cues are defined as an adult conveying to students, through their discourse, the expected norms for how to participate in the visual language conversation (see Table 4). As Smith and Ramsey (2004) documented in a fifth-grade classroom of deaf students, the deaf teacher invites students to participate in the teacher-directed group interaction, using signs like “NOW” or “OK,” conveying that it is time to be quiet and pay attention. In this classroom of deaf students, the teacher establishes individual gaze and/or point, nod, or uses a non-manual marker to a child to yield them the speaker’s role (Mather, 1987; Smith and Ramsey, 2004). Sometimes her hand will remain pointing to help other children “find” the child who now has the floor. This placeholder also conveys that other children should not interrupt. The children can also anticipate their upcoming turn when the teacher invites them with a sign like YOU-NEXT!

Successful participation in a visual language conversation also requires optimizing visual sightlines, ensuring that no obstacles or persons are obstructing their view of the teacher’s signing. For example, the teacher may issue a directive telling a child to alter their undesirable position, by signing phrases such as MOVE-BACK, MOVE-FORWARD, SIT-UP, and asking SEE CLEAR?

Participation cues also include teacher behaviors that have the effect of delaying or refusing a child’s bid for participation. For example, when a child tries to interrupt the teacher or another child who is signing (i.e., they hold the floor in the group conversation), the teacher tries to *delay* the child’s participation, by using signs like WAIT (index finger held up), HOLD, WILL++ (e.g., you will have your turn). Sometimes, when a child persistently tries to get the teacher’s attention (when the teacher is attending to another child), even after they have been asked to wait, the teacher will *refuse* their bid by purposely not looking at them or even pushing their “waving hand” down.

## Results

The purpose of this study is to examine the ways in which deaf teachers socialize deaf preschoolers into full participation in a visual language ecology. Our detailed classroom observations focus on one deaf teacher, and one deaf teacher’s aide, as they interact with six deaf preschoolers in two separate teacher-directed group instruction settings. The first episode, the “Picture-Walk” (21 m, 33 s), is considered less-structured and the preschoolers are allowed to freely participate in the communication interaction as they sit in a semi-circle facing the teacher who is “walking them through” a children’s picture book without explicitly reading it to them. The second episode, the “Role Play” (15 m, 40 s), is more structured than the first activity as each student is provided with an explicit participation turn (role play) in the story-retelling. Turn-taking in this activity is regulated by the teacher. This observation also involves a deaf teacher’s aide who is sitting behind the children in the semi-circle facing the teacher.

Our analysis for this study focuses on the attention actions and participation cues produced by both the teacher and the teacher’s aide. We count the number of prompts geared toward the whole group (as indicated by what Mather terms *group-directed gaze or audience gaze)* or toward individual students (*individual-directed gaze)*. These individual prompts are also divided according to whether they are directed toward Deaf children of Deaf parents (DoD), or deaf children of hearing parents (DoH). We are especially interested in whether the patterns of teacher behavior differ when they are directed toward DoD as compared to DoH. This comparison is of particular interest as we expect that DoD preschoolers at this age would already possess visual modality capital because of their experience in the home environment of being socialized early into a visual language ecology. Thus, we predict that DoD will less often be the target of attention-gaining or directing actions from the teacher compared to DoH students who are presumably entering the classroom (i.e., this developmental niche) with less prior visual language experience (i.e., less modality capital).

### Attention-gaining actions

The teacher uses Attention-Gaining actions to elicit the students’ attention either through linguistic prompts (e.g., handwaves, LOOK-AT-ME), physical prompts (e.g., light touches on the body), or non-manual prompts (e.g., raised eyebrows). Overall, we document a total of 187 Attention-Gaining (AG) prompts that the teacher directs to students in Episodes 1 and 2 combined. Of the 187 AG prompts, 109 (58%) are directed toward students who have hearing parents (DoH), 65 (35%) are directed toward students with deaf parents (DoD), and 13 prompts (7%) are directed toward the class as a whole. These results are summarized in Table 5.

**TABLE 5:** Teacher and teacher aide attention gaining actions in the unstructured picture walk (21:33) and structured role play (15:40) episodes.

Attention gaining	Tokens			Frequency		
	Group	DoD	DoH	Group	DoD	DoH
**Teacher (unstructured)**						
Linguistic prompt	3	26	20	0.06	0.53	0.41
Physical prompt	1	12	31	0.02	0.27	0.70
Non-manual prompt	2	0	1	0.67	0.00	0.33
Total	6	38	52	0.06	0.40	0.54
**Teacher (structured)**						
Linguistic prompt	4	7	12	0.17	0.30	0.52
Physical prompt	2	16	8	0.03	0.62	0.31
Non-manual prompt	1	2	2	0.20	0.40	0.40
Total	7	25	22	0.13	0.46	0.41
**Teacher aide (structured)**						
Linguistic prompt	0	2	3	0.00	0.40	0.60
Physical prompt	0	0	32	0.00	0.00	1.00
Non-manual prompt	0	0	0	0.00	0.00	0.00
Total	0	2	35	0.00	0.05	0.95
Overall total	13	65	109	0.07	0.35	0.58

The overall results indicate similarities and differences in the types of Attention Gaining prompts geared toward the DoH and DoD students. The two groups of students receive the same number of linguistic prompts (*n* = 35) and a similar number of non-manual prompts (*n* = 6 and *n* = 4, respectively) directed toward them. While the non-manual prompts are used sparingly as an isolated directive (e.g., raised eyebrows), this nevertheless appears to be a subtle tool used to gain the student’s attention.

In contrast, the DoH students receive far more physical prompts (71 out of 102) from the teacher than do the DoD students (28 out of 102) even though the distance to reach any student is essentially equal as they are positioned in a semi-circle in front of her. This difference is illustrated by the fact that the teachers often resort to a physical touch to get the attention of the DoH students, especially if they are unable to get their attention through the discourse-embedded strategies of linguistic or non-manual prompts.

In comparing the two episodes, when the teacher’s aide is present (in the Structured activity), the teacher lessens her use of the physical prompts, seemingly relegating that responsibility to the aide (Note: we observed on the video the teacher asking the aide to sit near the three DoH students to “help manage them”). Specifically, in the Picture Walk (Episode 1), the teacher directs more physical prompts toward the DoH students (*n* = 31) than the DoD students (*n* = 12). In the Role Play (Episode 2), the teacher and aide combined direct 40 physical prompts toward the DoH students compared to only 16 toward the DoD.

### Attention-directing actions

The teachers appear to use the Attention-Directing (AD) actions to help students focus their attention on the primary person (e.g., teacher or student) or object of interest. As summarized in Table 6, of the 43 AD actions documented in both episodes, by both teacher and aide, 42 are linguistic prompts (e.g., LOOK-there) and one prompt is physical (the aide touches an object that a student was holding). This makes sense because (as was reported to us by several deaf teachers) within Deaf Culture one would not normally rely upon a physical prompt to redirect the child’s attention (i.e., it would be rude to place one’s hand on a person’s head and forcibly turn it toward the new target). In total, 86% of the AD actions produced by the teachers are directed toward the DoH students (*n* = 37), while the DoD receive only 12% (*n* = 5). Only one linguistic AD prompt (2%) is directed toward the class as a whole. Due to the nature of the activity, the teacher uses the Attention Direct prompts sparingly, as she is focused mostly on gaining their attention (to herself) and eliciting information from the students. By comparison, as is appropriate for her role, the teacher’s aide makes far greater use of the Attention Direct prompts (e.g., LOOK-AT TEACHER!) to scaffold the direction of the DoH students’ gaze.

**TABLE 6:** Teacher and teacher aide attention directing actions in the unstructured picture walk (21:33) and structured role play (15:40) episodes.

Attention directing	Tokens			Frequency		
	Group	DoD	DoH	Group	DoD	DoH
**Teacher (unstructured)**						
Linguistic prompt	0	3	4	0.00	0.43	0.57
Physical prompt	0	0	0	0.00	0.00	0.00
Non-manual prompt	0	0	0	0.00	0.00	0.00
Total	0	3	4	0.00	0.43	0.57
**Teacher (structured)**						
Linguistic prompt	0	1	2	0.00	0.33	0.67
Physical prompt	0	0	0	0.00	0.00	0.00
Non-manual prompt	0	0	0	0.00	0.00	0.00
Total	0	1	2	0.00	0.33	0.67
**Teacher aide (structured)**						
Linguistic prompt	1	1	30	0.03	0.03	0.94
Physical prompt	0	0	1	0.00	0.00	1.00
Non-manual prompt	0	0	0	0.00	0.00	0.00
Total	1	1	31	0.03	0.03	0.94
Overall total	1	5	37	0.02	0.12	0.86

**FIGURE 1:**
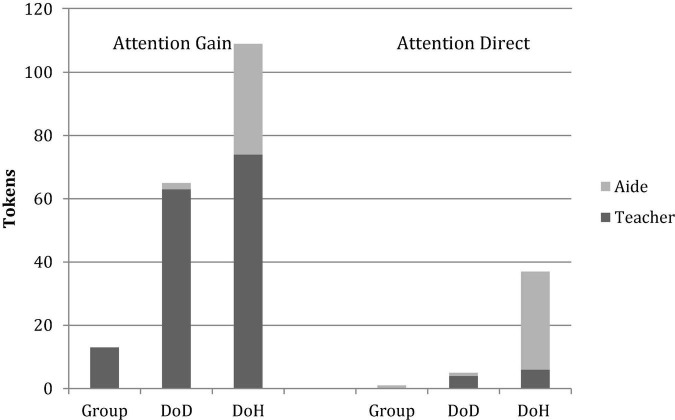
Deaf preschool teachers’ overall tokens (collapsed across two book-sharing episodes) of Attention-Gaining and Attention-Directing Actions expressed toward the whole group of students, Deaf children of Deaf parents (DoD) and deaf children of hearing parents (DoH).

Figure 1 illustrates the combined pattern of results presented in Tables 5, 6.

### Participation cues

**FIGURE 2:**
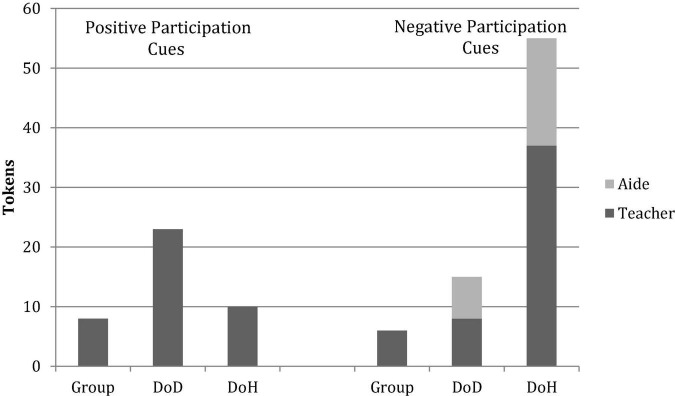
Deaf preschool teachers’ overall (collapsed across two book-sharing episodes) positive and negative participation cues expressed toward the whole group of students, Deaf children of Deaf parents (DoD), and deaf children of hearing parents (DoH).

The participation cues are divided into types of cues that appear to encourage students’ positive participation like READY? or YOU-NEXT! (Invite) with those that discourage negative behaviors such as interruptions (Directive, Delay, Refusal). The results of this analysis are summarized in Table 7 and Figure 2, with details reported in the following section.

**TABLE 7:** Teacher and teacher’s aide participation cues in the unstructured picture walk (21:33) and structured role play (15:40) episodes.

Participation cues	Tokens			Frequency		
	Group	DoD	DoH	Group	DoD	DoH
**Teacher (unstructured)**						
**Positive**						
Invite	5	14	5	0.21	0.58	0.21
**Negative**						
Directive	4	1	8	0.31	0.08	0.62
Delay	0	0	1	0.00	0.00	1.00
Refuse	0	0	16	0.00	0.00	1.00
Negative total	4	1	25	0.13	0.03	0.83
**Teacher (structured**)						
**Positive**						
Invite	3	9	5	0.18	0.53	0.29
**Negative**						
Directive	0	3	11	0.00	0.21	0.79
Delay	2	1	0	0.67	0.33	0.00
Refuse	0	3	1	0.00	0.75	0.25
Negative total	2	7	12	0.09	0.33	0.57
**Teacher aide (structured)**						
**Positive**						
Invite	0	0	0	0.00	0.00	0.00
**Negative**						
Directive	0	0	10	0.00	0.00	1.00
Delay	0	1	8	0.00	0.11	0.89
Refuse	0	0	0	0.00	0.00	0.00
Negative total	0	7	18	0.00	0.05	0.95
Overall positive total	8	23	10	0.20	0.56	0.24
Overall negative total	6	15	55	0.08	0.20	0.72

In terms of positive participation cues produced by the teachers, a total of 41 Invites are documented across both episodes. The DoD students receive 23 Invites (56%), compared to 10 Invites for DoH (24%), and eight are offered to the whole group (20%). The teacher’s pattern of Invites seems to vary by context. The Picture Walk is much more unstructured, and the teacher noticeably directs more of her Invites toward individual DoD students (*n* = 14) compared to DoH (*n* = 5) students and the whole class (*n* = 5). This pattern may reflect a higher level of language abilities possessed by the DoD, and the fact that they are reliably visually engaged, as compared to the DoH students. The DoD students often provide more elaborated responses to the teacher’s question, while DoH students more typically give minimal (one word) responses, to which the teacher consistently expanded upon or asked follow-up questions to elicit further information.

In the more structured episode, the Role Play, each student has an assigned role and turns are negotiated by the teacher; this structure limits the opportunity for students to contribute spontaneously. As a result, the DoD receive comparably fewer invites (*n* = 9) than the unstructured episode (*n* = 14), while the invite number for the DoH (*n* = 5) remains the same across episodes.

In terms of participation cues to discourage students’ negative behavior, the DoH receive a noticeably larger number of corrective prompts from the teachers. Across the two episodes, a total of 76 negative cues are documented (Directive, Delay, and Refusal); among these, the DoH receive 55 prompts (72%), the DoD receive 15 prompts (20%), and the whole class receives six prompts (8%). Across the two episodes, the DoH students receive a similar number of Directives from the teacher (about 10 per episode); however, an additional 10 Directives are issued to the DoH by the aide during the Role Play episode. By contrast, the DoD receive a total of only five such prompts across the two episodes from both teachers. Similarly, the DoH receive more Delay actions (*n* = 9) from the teacher and the aide, as compared to the DoD (*n* = 2). This was especially evident when the students are bidding for characters in the beginning of the Role Play activity.

Likewise, the DoH students are the primary recipients of the Refusal cues used by the teacher. The teacher conveys her refusal to yield the floor by refusing to grant eye contact to student(s) who are deliberately waving or physically touching her while she is signing to another student, looking up information in a book, or attempting to distribute a prop to a student. Across both activities, there are a total of 20 occurrences where the teacher refuses a student’s inappropriate bid for attention. Of the 20 occurrences, 17 involve DoH students (85%), and three involve DoD students (15%). Most of these Refusals occur during the unstructured Picture Walk activity (16 of 20), when the DoH students seem to be less able to navigate the turn-taking appropriately without the clear cues provided by the structured episode.

## Discussion

This classroom observational study focuses on how a Deaf preschool teacher and her teacher’s aide promote the development of visual modality capital (gaining and directing attention) and a visual language ecology (through participation cues) with their deaf preschool-aged students. We note that across the two book-sharing contexts we observed, all the preschoolers are frequently prompted by the teacher with linguistic reminders that they should be paying attention. However, two further insights emerge from these observations. First, the teachers’ seemingly different behavior toward Deaf children of Deaf parents (DoD) and deaf children of hearing parents (DoH) provides compelling evidence that by age four DoD are well on their way to possessing visual modality capital through self-control over their visual attention and understanding the turn-taking expectations of a visual language conversation. In many cases, the DoD only need the teacher’s more subtle positive participation cue (e.g., “READY?”) to alert them that it is time to pay attention. Furthermore, as evidenced by the teacher’s increased use of explicit attention socialization strategies toward them, the DoH children appear to still be developing their visual modality capital. The DoH preschoolers are on the receiving end of more attention-socializing directives that are heavily dependent upon physical prompts and corrective prompts likely in response to inattention or inappropriate bids for attention.

Based on the differential interactions between the teachers in this study and their DoD vs. DoH students, we suggest that DoD preschoolers arrive to the classroom with well-established visual modality capital, likely because they have been raised within a developmental niche that promotes visual engagement and self-regulation of attention. This aligns with Spencer et al.’s (1992) observation that between 9 and 18 months of age, deaf caregivers first use physical tapping to attract their deaf infant’s attention and then shift to using more linguistic cues as their child approaches 18 months of age. Spencer et al. found that as they became older DoD children would anticipate their caregiver’s signing without needing explicit cueing from their caregiver. While we do not specifically code the student’s looking patterns, the teachers’ actions suggest that the DoD children do not need physical cueing because they are already following the teacher’s signing or anticipating her directive on where to look. With the DoH children in our study, it appears that the teachers may be mirroring the kind of socialization patterns observed in Deaf caregiver-infant dyads in that they deploy physical prompts to gain and maintain their attention (in parallel with linguistic cues), presumably because they are responding to failures of looking appropriately or interruptions to turn-taking expectations.

Lieberman (2015) found that native ASL signing preschoolers (age 19–39 months) also use the same attention-getting strategies that we document in our deaf teachers (tapping, waving). It is impressive that at such a young age, these children’s initiations with their peers are successful roughly two-thirds of the time. Lieberman states that these young children are already “aware of the need to establish eye contact with their interlocutors in order to communicate in the visual mode” (10) and suggests that they are generalizing from how they have been socialized at home to their classroom interactions with their peers.

A second interesting finding emerges with respect to the nature of the classroom activity. One activity (Picture Walk) is more unstructured, while the other (Role Play) has well-defined, predictable turns for the children to take. Because the unstructured activity likely increases the self-regulation and communication burden on the child, the more-skilled DoD have a clear advantage over the DoH students. In this setting, the DoH frequently interrupt the teacher and needed to be directed more often. By contrast, the Role Play activity is more structured with predictable turn-taking patterns. Here, we do not observe the DoH children interrupting; however, the teacher and the teacher’s aide are still fairly directive toward the DoH seemingly to help them keep on task and support their engagement in the structured activity.

Lastly, we note that the teacher frequently repeated her instructions. While it is not within the scope of this analysis, we do feel that further research is needed on why a signing teacher may be repeating her utterances so much (our intuition says this repetition was even more than what “preschool teacher register” would engender). We know from Pizer et al.’s (2011) study with infants and young toddlers that deaf caregiver repetitions occur even when the child’s gaze was connected (that is, that their repetition was not due to the child missing the caregiver’s signing because of inattention). Pizer et al. (2011) suggest that caregiver repetitions may be an invitation to the child to imitate or respond. In the case of our preschool classroom with six children closer to age five, the teacher’s repetition of signing may serve to accommodate a child who has missed the teacher’s signing through inattention, or it may be a characteristic of a child-directed language register that she intuitively deploys knowing that half of the students in her class are still acquiring ASL and still establishing visual modality capital.

## Conclusion and implication for practice

Based on the results from this in-depth observation of teacher-student interaction, our study suggests that, at least for this Deaf teacher and her aide, socialization patterns for promoting student’s visual modality capital are reminiscent of how Deaf caregivers engage with their deaf children in infancy. Overall, like Lieberman (2015), we observe that the Deaf preschoolers from Deaf parented families seemed to already know how to engage visually and are thus “ready to learn” and appear to respond well to the teacher’s explicit and implicit (linguistic, discourse-embedded) attention prompts that support interaction in a visual language [see Horton and Singleton (2022) this volume, for a review of turntaking practices in a signed language]. These DoD students are more frequently invited to participate because it appears that they anticipated the teacher’s invitation (i.e., they were already looking at her when she was doing the inviting and could thus appropriately respond). For the DoH preschoolers, who appear to be still developing their self-regulation of attention capacity (i.e., leveraging visual modality capital for language acquisition), the teacher and her aide more often use physical prompts (such as a physical tap) to attract and direct their attention because, based on our observation, it appears that the students have not visually anticipated her invitation to participate.

As this is only a single observational study, we are careful about broader generalizations that could be made from our observations; even so, we do offer a few ideas for classroom implications based on our findings and those of others. Teachers, or teacher’s aides, may want to sit close enough to emergent signers so that they can use a physical touch to alert them to attend. Gradually, or even in parallel, a teacher could increase their use of linguistic prompts, and decrease the use of physical signals, to promote the child’s self-regulation of attention.

Structured group participation activities can also help a deaf child engage with their teacher and peers in visually predictable ways (e.g., following a fixed order for activities that require individual turns). Still, it would be important to gradually mix in more unstructured activities to give children increasing experience with spontaneously requesting a bid for attention, holding the floor, and rapidly shifting their gaze to other conversation participants.

Finally, activities that require children to shift their gaze amongst a series of visual targets may help promote their visual modality capital. For example, a child might be expected to shift their attention between the signing teacher, a large flipchart, and a collection of illustrations (e.g., pictures of farm animals) to be selected from (for putting on the chart). Also, it might be useful to ask two linguistic models to share the storytelling in a book reading event so that the children must shift their attention between two narrators and the book.

While this study offers an in-depth look at deaf teacher-deaf student interaction using a visual modality lens, we recognize that it is based on a sample drawn from a limited context (an ASL-using school with Deaf preschool teachers). In the future, it will be important to examine visual language ecologies across a broader range of structured and unstructured educational contexts, including children with different language and modality experiences (e.g., deaf children with cochlear implants, hearing children of deaf parents acquiring both English and ASL), and from different cultural settings where gaze norms may vary significantly from the US context that we explored here. In addition, it is important to examine the role of other skilled signers besides the teacher in helping a novice strengthen their visual attention and language skills. Lieberman’s (2015) study of native ASL signing deaf toddlers (ages 21–39 months) in a preschool provides ample evidence that children even this young use attention-getting strategies to engage with their peers.

Considering “developmentally appropriate” or “best practices” in early childhood education in more general terms, we recognize that the field would not necessarily advocate for a heavy reliance on “teacher-centered” group-based instruction, favoring instead free-choice, center-based, discovery-type learning. In the context of deaf education, however, it may be the case that a child draws different benefits from group instruction or teacher-mediated interaction especially because such contexts provide greater demand within the visual modality insofar as these discourse frameworks require increased attention shifting and anticipatory looking on the part of the child.

A final point to emphasize here is that we conceptualize the promotion of a deaf child’s visual modality capital by immersing them in a natural visual language and *scaffolding their visual engagement*. Like Dye et al. (2008), we do not feel that “stripping down” a child’s visual world or eliminating all visual distractions (e.g., placing them in the front of the class or setting up physical barriers to reduce visual access to background distractions) is an ecologically valid approach to strengthening their visual modality capital [in fact, Dye et al. (2008) argue that such arrangements may even exacerbate the situation]. Because deaf individuals have adapted their visual systems to maintain vigilance in attending to their periphery while attending to a central point of focus (Proksch and Bavelier, 2002), it is the *unexpected* visual distractions in the periphery that appear to be the most intrusive. Dye et al. (2008) suggest that we allow a child *to learn* to navigate the expected level of “visual noise” and adapt to the visual demands of their learning environment. By structuring their visual modality capital, we *increase the predictability* of their visual language interactions, which may subsequently reduce their sensitivity to peripheral distractions. We would also argue that to strengthen the ecological validity of this “structuring approach” we must look to how Deaf parents and Deaf teachers have routinely solved this challenge vis-à-vis their “indigenous practices” (Humphries, 2004) or intuitive practices (Papoušek and Papoušek, 1987). By applying these culturally- and modality-appropriate environmental supports for language and visual modality socialization in the classroom, teachers can create developmental niches that unlock intuitive adaptations for learners who are deaf and who learn language through the visual modality.

## Data availability statement

The video source datasets presented in this article are not readily available as they contain identifiable information about participants. Requests to access de-identified datasets should be directed to JS, jenny.singleton@stonybrook.edu.

## Ethics statement

The studies involving human participants were reviewed and approved by University of Illinois at Urbana-Champaign. Written informed consent to participate in this study was provided by the participants (teachers) or their legal guardian/next of kin (children).

## Author contributions

JS designed the study, collected the data, participated in data coding and analysis, and led the writing of the manuscript. PC conducted data coding and analyses and significantly contributed to the writing of the manuscript. Both authors contributed to the article and approved the submitted version.

## Footnotes

1 Horton, L., Hou, L., German, A., and Singleton, J. (under review). *Sign Language Socialization and Participant Frameworks in Three Indigenous Mesoamerican Communities*.
